# Clinical Courses of Two Pediatric Patients with Acute Megakaryoblastic Leukemia Harboring the CBFA2T3-GLIS2 Fusion Gene

**DOI:** 10.4274/tjh.2016.0008

**Published:** 2016-12-01

**Authors:** Mayu Ishibashi, Tomoko Yokosuka, Masakatsu D. Yanagimachi, Fuminori Iwasaki, Shin-ichi Tsujimoto, Koji Sasaki, Masanobu Takeuchi, Reo Tanoshima, Hiromi Kato, Ryosuke Kajiwara, Fumiko Tanaka, Hiroaki Goto, Shumpei Yokota

**Affiliations:** 1 Yokohama City University Faculty of Medicine, Department of Pediatrics, Yokohama, Japan; 2 Kanagawa Children’s Medical Center, Clinic of Hematology/Oncology and Regenerative Medicine, Yokohama, Japan

**Keywords:** Acute megakaryoblastic leukemia without Down syndrome, CBFA2T3-GLIS2 fusion gene

## Abstract

Acute megakaryoblastic leukemia (AMKL) in children without Down syndrome (DS) has an extremely poor outcome with 3-year survival of less than 40%, whereas AMKL in children with DS has an excellent survival rate. Recently, a novel recurrent translocation involving CBFA2T3 and GLIS2 was identified in about 30% of children with non-DS AMKL, and the fusion gene was reported as a strong poor prognostic factor in pediatric AMKL. We report the difficult clinical courses of pediatric patients with AMKL harboring the CBFA2T3-GLIS2 fusion gene.

## Abstract (tr)

Down sendromu (DS) olmayan çocuklarda akut megakaryoblastik löseminin (AMKL) prognozu çok kötü ve 3 yıllık sağkalım %40’ın altında iken, DS’li çocuklarda AMKL’nin sağkalım oranı mükemmeldir. Yakın zamanda, DS olmayan AMKL’li çocukların yaklaşık %30’unda CBFA2T3 ve GLIS2’yi içeren yeni bir tekrarlayan translokasyon tanımlandı ve füzyon geninin pediatrik AMKL olgularında kötü prognoz ile ilişkili güçlü bir prognostik belirteç olduğu bildirildi. CBFA2T3-GLIS2 füzyon genini taşıyan AMKL tanılı pediatrik hastalarda sorunlu klinik seyri bildiriyoruz.

## INTRODUCTION

Acute megakaryoblastic leukemia (AMKL) is classified as M7 in the FAB (French-American-British) classification. AMKL accounts for approximately 10% of pediatric acute myeloid leukemia (AML) cases and 1% of adult AML cases [1,2,3]. Pediatric AMKL is divided into two subgroups: AMKL arising in patients with Down syndrome (DS-AMKL), and AMKL arising in patients without DS (non-DS-AMKL). Although patients with DS-AMKL have an excellent survival rate, patients with non-DS-AMKL have an extremely poor outcome with 3-year survival of less than 40% [1,2,4]. Recently, two studies identified a novel recurrent translocation involving CBFA2T3 and GLIS2 in about 30% of children with non-DS-AMKL. The CBFA2T3-GLIS2 fusion gene was reported as a strong poor prognostic factor in pediatric AMKL [5,6]. We report the difficult clinical courses of two pediatric patients with AMKL harboring the CBFA2T3-GLIS2 fusion gene.

## CASE PRESENTATION

Between 2003 and 2012, six patients were diagnosed with AMKL at the Department of Pediatrics of Yokohama City University Hospital. We analyzed the fusion gene, CBFA2T3-GLIS2, in the six leukemic samples at the time of diagnosis by reverse transcription polymerase chain reaction (PCR) and direct sequencing, according to a previous report [5]. We compared characteristics between the patients who were diagnosed with AMKL with or without the CBFA2T3-GLIS2 fusion gene.

**Figure 1:**
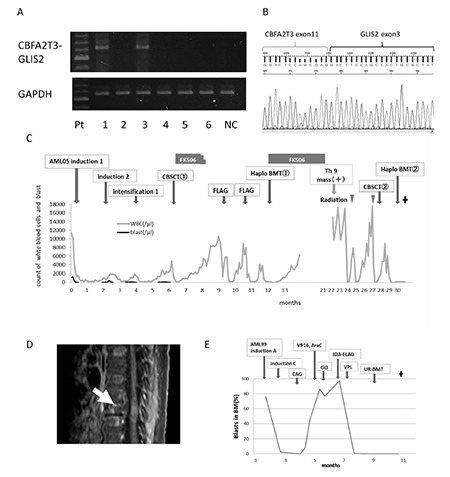
Clinical courses of two Acute megakaryoblastic leukemia patients with the CBFA2T3-GLIS2 fusion gene. A) Reverse transcription polymerase chain reaction for the CBFA2T3-GLIS2 fusion gene in our patients. Two patients with non-Down syndrome-acute megakaryoblastic leukemia (patients 1 and 3) had the CBFA2T3-GLIS2 fusion gene. NC: Negative control. B) Direct sequencing for the polymerase chain reaction product of the CBFA2T3-GLIS2 fusion gene in patient 1 revealed that exon 11 of CBFA2T3 was fused to exon 3 of GLIS2. C) Clinical course of patient 1. FLAG: Fludarabine, cytarabine, G-CSF; FK506: tacrolimus. D) Magnetic resonance imaging of patient 1 revealed an extramedullary lesion at the thoracic spinal cord (Th9). E) Clinical course of patient 3. CAG: Cytarabine, aclarubicin, G-CSF; GO: gemtuzumab ozogamicin; IDA: idarubicin; VPL: vincristine, prednisolone, L-asparaginase.

Two patients had DS-AMKL harboring a GATA1 mutation and four had non-DS-AMKL. None of them had inv(16)/t(16;16) chromosomal abnormalities upon G-band karyotyping. Two patients with non-DS-AMKL (Patient 1 and Patient 3) had the CBFA2T3-GLIS2 fusion gene (Table 1). Reverse transcription PCR and direct sequencing revealed that exon 11 of CBFA2T3 was fused to exon 3 of GLIS2 in both cases (Figures 1A and 1B). Neither of them achieved complete remission (CR) after induction therapies. They died from the primary disease after stem cell transplantation (SCT). The other 4 patients remain alive in CR (Table 1).

Patient 1 with the CBFA2T3-GLIS2 fusion gene was treated under the AML05 protocol of the Japanese Pediatric Leukemia/Lymphoma Study Group [7] and could not achieve CR after induction 1 therapy (Figure 1C). After induction 2 therapy, Patient 1 under non-CR conditions was treated with unrelated cord blood SCT (CBSCT) after a myeloablative conditioning regimen. Three months after CBSCT, her AMKL relapsed. She underwent two courses of chemotherapy. She received a haploidentical SCT (haplo-SCT) from her mother under non-CR conditions. After the second transplant, she had leg paralysis and bladder and rectal disturbance from an extramedullary lesion at the thoracic spinal cord (Th9) (Figure 1D). Although she underwent radiation therapy for the Th9 mass, the mass did not disappear. While she received a second CBSCT and haplo-SCT, she failed to engraft and died 30 months after the fourth SCT.

Patient 3 with the CBFA2T3-GLIS2 fusion gene was treated under the AML99 protocol [8] and could not achieve CR after induction A therapy (Figure 1E). She did not achieve CR even after several types of chemotherapy. Thereafter, she underwent chemotherapy with vincristine, prednisolone, and L-asparaginase (VPL), which is commonly used in therapy for acute lymphoblastic leukemia (ALL). After the VPL therapy, the percentage of blastic cells in the bone marrow decreased. She received unrelated bone marrow transplantation after a reduced-intensity conditioning regimen. She maintained remission for about 180 days and thereafter relapsed. Despite treatment with drugs including imatinib and L-asparaginase, she died 23 months after bone marrow transplantation.

## DISCUSSION

It was reported that CBFA2T3-GLIS2 fusion gene-positive cases account for about 30% of pediatric patients with AMKL [5,6,9]. In addition, the overall survival rate and the event-free survival rate were lower in patients with the CBFA2T3-GLIS2 fusion gene than in those without this fusion gene [5,9,10,11]. There is little information about the clinical course of these patients. We encountered two AMKL patients with poor prognostics harboring the CBFA2T3-GLIS2 fusion gene, even though neither of them had inv(16)/t(16;16) chromosomal abnormalities upon G-band karyotyping. Therefore, evaluation of AMKL patients with this fusion gene without inv(16)/t(16;16) is needed.

CD56 was expressed in leukemic blasts of the two CBFA2T3-GLIS2-positive patients with AMKL but not in the two CBFA2T3-GLIS2-negative patients among the non-DS-AMKL patients in our cohort (Table 1). It was reported that CD41 and CD56 were positive and CD56 was drastically more highly expressed in patients with CBFA2T3-GLIS2-positive AMKL [6]. Higher expression of the CD56 antigen was reported as a poor prognostic marker [9,12,13,14,15,16,17]. Some investigators demonstrated that patients with CD56 positivity in blasts showed a higher incidence of extramedullary manifestations [12,13,14,18]. Among our patients with AMKL, CD56 was also more highly expressed in the two CBFA2T3-GLIS2-positive patients with AMKL with poor outcomes, and Patient 1 had extramedullary manifestation that did not regress after irradiation. High CD56 expression may be a surrogate marker of CBFA2T3-GLIS2 positivity in AMKL.

In Patient 3 with CBFA2T3-GLIS2-positive AMKL, chemotherapy regimens used to treat AML were not effective, but chemotherapy with VPL, commonly used to treat ALL, seemed to be more effective. When some of the treatment strategies commonly used to treat AML are not effective, the type of chemotherapy used to treat ALL might be effective in a subpopulation of patients with AMKL. There is a possibility that the conventional treatment commonly used to treat ALL may be effective for AMKL with this fusion gene. Eventually, the AMKL in both of the CBFA2T3-GLIS2-positive patients in our cohort became intractable to treatment, including SCT. Despite some chemotherapy regimens and SCT, the two patients with the CBFA2T3-GLIS2 fusion gene had poor prognosis. As previously reported, CBFA2T3-GLIS2 expression enhances BMP2/BMP4 signaling [5]. The development of treatments including novel targeted therapy drugs is desired.

## CONCLUSION

Clinical courses of pediatric patients with AMKL harboring the CBFA2T3-GLIS2 fusion gene are poor due to resistance to chemotherapies and SCT. New treatment strategies are necessary.

## Ethics

Ethics Committee Approval: The protocol of this survey and research plan has been approved by the Clinical Ethics Committee of Yokohama City University (A130725002), Informed Consent: It was taken from patients and/or their parents.

## Figures and Tables

**Table 1:**
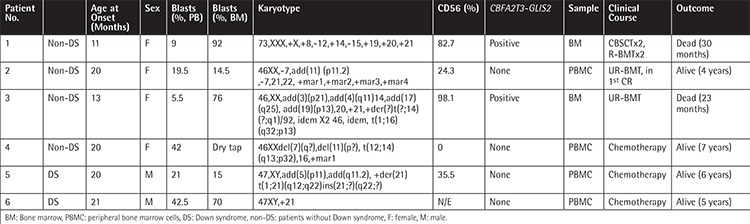
Patient details.
